# Myeloperoxidase mediated HDL oxidation and HDL proteome changes do not contribute to dysfunctional HDL in Chinese subjects with coronary artery disease

**DOI:** 10.1371/journal.pone.0193782

**Published:** 2018-03-05

**Authors:** Guisong Wang, Anna Vachaparampil Mathew, Haiyi Yu, Lei Li, Liyun He, Wei Gao, Xiaodan Liu, Yanhong Guo, Jaeman Byun, Jifeng Zhang, Y. Eugene Chen, Subramaniam Pennathur

**Affiliations:** 1 Department of Cardiology and Institute of Vascular Medicine, Peking University Third Hospital, Beijing, China; 2 Key Laboratory of Cardiovascular Molecular Biology and Regulatory Peptides, Ministry of Health, Beijing, China; 3 Key Laboratory of Molecular Cardiovascular Science, Ministry of Education, Beijing, China; 4 Beijing Key Laboratory of Cardiovascular Receptors Research, Beijing, China; 5 Department of Internal Medicine, University of Michigan, Ann Arbor, Michigan, United States of America; 6 Department of Medicine, Nephrology, First Affiliated Hospital, China Medical University, Shenyang, China; Nagoya University, JAPAN

## Abstract

High density lipoprotein (HDL) cholesterol levels and cholesterol efflux capacity (CEC) are inversely correlated with coronary artery disease (CAD) risk. Myeloperoxidase (MPO) derived oxidants and HDL proteome changes are implicated in HDL dysfunction in subjects with CAD in the United States; however, the effect of MPO on HDL function and HDL proteome in ethnic Chinese population is unknown. We recruited four matched ethnic Chinese groups (20 patients each): subjects with 1) low HDL levels (HDL levels in men <40mg/dL and women <50mg/dL) and non-CAD (identified by coronary angiography or cardiac CT angiography); 2) low HDL and CAD; 3) high HDL (men >50mg/dL; women >60mg/dL) with no CAD; and 4) high HDL with CAD. Serum cytokines, serum MPO levels, serum CEC, MPO-oxidized HDL tyrosine moieties, and HDL proteome were assessed by mass spectrometry individually in the four groups.

The cytokines, MPO levels, and HDL proteome profiles were not significantly different between the four groups. As expected, CEC was depressed in the entire CAD group but more specifically in the CAD low-HDL group. HDL of CAD subjects had significantly higher 3-nitrotyrosine than non-CAD subjects, but the MPO-specific 3-chlorotyrosine was unchanged; CEC in the CAD low-HDL group did not correlate with either HDL 3-chlorotyrosine or 3-nitrotyrosine levels. Neither 3-chlorotyrosine, which is MPO-specific, nor 3-nitrotyrosine generated from MPO or other reactive nitrogen species was associated with CEC. MPO mediated oxidative stress and HDL proteome composition changes are not the primary cause HDL dysfunction in Chinese subjects with CAD. These studies highlight ethnic differences in HDL dysfunction between United States and Chinese cohorts raising possibility of unique pathways of HDL dysfunction in this cohort.

## Introduction

Cardiovascular disease (CVD) mortality from stroke and coronary artery disease (CAD) continues to be the primary public health issue in China [1, 2]. CVD risk factors including high blood pressure, high salt intake, smoking exposure, physical inactivity, and low high density lipoprotein (HDL) cholesterol levels are highly prevalent in China but do not entirely explain CVD risk [3, 4]. Waist circumference, geographic location, urbanization, and family history of CVD significantly improved the CVD risk prediction model built specifically for the Chinese population compared to traditional prediction models [5]. Applying this risk model to Caucasian subjects in United States (US), or applying the Framingham risk score to Chinese, yielded very different predictive results, underscoring the presence of unique risk factors in both populations that are not transposable. Environmental factors (e.g., geographic region and urbanization) and familial aggregation (either because of shared environmental factors or genetic determinants of risk factors) play an essential role in Chinese CVD risk. This unique risk factor burden in the Chinese population underscores the need to explore the mechanism behind these differences.

Epidemiologic studies and large-scale clinical trials have conclusively established a strong inverse relationship between HDL levels and CVD risk in both the Western and Chinese populations [6–8]; however, the influence of HDL levels on CVD pathogenesis has recently been called into question, especially when pharmacological attempts to raise HDL levels fail to improve cardiovascular outcomes [9]. In fact, low HDL levels are specific to CVD mortality, while higher non-CVD mortality is associated with elevated HDL levels [10]. HDL levels are merely one dimension of HDL’s myriad functions that include cholesterol efflux, anti-oxidative, anti-inflammatory, anti-apoptotic, and anti-coagulant properties [11]. Importantly, decreased cholesterol efflux capacity (CEC) has been found to predict CVD independent of HDL levels [12, 13] and paradoxically increased efflux associated with poor prognosis and CAD incidence in US patients [14]. We and others have shown that HDL proteome modification alters HDL function and is associated with CVD risk [15–18]. Thus, HDL function and its protein composition are associated with CAD risk in addition to HDL levels alone.

Oxidative stress, specifically HDL oxidation and subsequent dysfunction, has been implicated in CVD. One well-characterized source of oxidative stress is myeloperoxidase (MPO), a heme enzyme that co-localizes with macrophages in human atherosclerotic lesions. High blood levels of MPO are associated with established CVD and identify individuals at increased risk for CAD and cardiovascular events [19–21]. HDL is a primary target for oxidative modification in patients with established CAD. HDL isolated from aortic lesions in CAD patients contains high levels of 3-chlorotyrosine and 3-nitrotyrosine, two abnormal oxidized amino acids that are characteristic of MPO activity [22, 23]. HDL oxidation selectively impairs the lipoprotein’s ability to remove cholesterol from cells, rendering the lipoprotein atherogenic [15, 24].

Inflammation and oxidative stress are particularly relevant to the Chinese population because distinct non-traditional pathways seem to be at play. In current clinical practice, biomarkers are limited by their lack of specificity in identifying critical pathogenic pathways and, for our purposes, their ability to define specific subsets of patients prone to developing CAD in China. Therefore, there is a pressing need for mechanism-based novel biomarkers that can accurately stratify CAD risk in ethnic Chinese patients above and beyond the traditional Framingham paradigm. We hypothesized that MPO-oxidized, dysfunctional HDL and its protein cargo are associated with CAD risk in Chinese patients irrespective of HDL levels. In this study, we utilized stored plasma samples from the Peking University Health Sciences Center (PUHSC) Cardiovascular study that recruited well-matched patients with CAD and without CAD (non-CAD) with high and low HDL levels. We assessed all subjects’ CEC at baseline and examined the relationship between oxidized HDL, levels of MPO, HDL proteome, and CEC. We found that HDL is dysfunctional in CAD patients, but MPO and other reactive nitrogen species (RNS) do not mediate the dysfunction. Our study is the first to highlight these significant ethnic differences between US and Chinese cohorts; thus, it may facilitate the development of therapies directed at these mechanistic differences from the Western population.

## Material and methods

### Study design and patient population

This cross-sectional case-control study enrolled a total of 80 subjects from PUHSC between May 2012 and June 2014. Four groups of subjects (20 patients per group) were recruited: a) non-CAD subjects with low HDL; b) CAD subjects with low HDL; c) non-CAD subjects with high HDL; and d) CAD subjects with high HDL. The low-HDL group was defined as men with HDL levels <40mg/dL and women with HDL levels <50mg/dL. The high-HDL group was defined as men with HDL levels >50mg/dL and women with HDL levels >60mg/dL. CAD subjects were identified using coronary angiography or cardiac Computed tomography (CT) angiography at entry to the study. Clinical data and blood samples were collected from all subjects after obtaining informed consent.

Inclusion criteria were as follows: subjects between 30–75 years old; in those with CAD, coronary stenosis ≥50% confirmed in at least one of the main branches of the coronary arteries as indicated by coronary angiography and/or coronary artery CT; in those without CAD, normal coronary arteries or only irregularities or coronary stenosis ≤20% in the main branches of the coronary arteries as confirmed by coronary angiography and/or coronary artery CT.

Exclusion criteria included the following: subjects with acute myocardial ischemia precipitated by atherosclerotic disease or any condition other than atherosclerotic coronary artery disease (e.g., arrhythmia, severe anemia, hypoxia, thyrotoxicosis, cocaine use, severe valvular disease, or hypotension); history of previous myocardial infarction; history of diabetes mellitus; left ventricular ejection fraction (<30%) or end-stage congestive heart failure (NYHA III or IV); renal dialysis or severe chronic kidney disease with plasma creatinine >2mg/dL or estimated glomerular filtration rate (Modification of Diet in Renal Disease) <30 mL/min/1.73 m^2^ or undergoing hemodialysis or peritoneal dialysis[25]; active liver disease or hepatic dysfunction, as determined by alanine aminotransferase (ALT) >3.0 x upper limit of normal (ULN)or bilirubin levels >1.5 x ULN at screening; use of statins within the past 3 months; and pregnant or lactating women.

At the screening visit, the investigators completed the study eligibility criteria checklist, obtained a complete medical history, and performed a general medical and focused physical exam. All subjects underwent laboratory testing, including a comprehensive blood panel and fasting lipid profile. Subjects also underwent coronary angiography or cardiac CT angiography before enrollment to be categorized into the CAD or non-CAD group. If the patient met the study criteria, the first visit consisted of a complete physical examination with detailed anthropometric parameters, standardized blood pressure measurement, and blood collection for measurements of fasting glucose, insulin levels, and oxidative stress. All subjects signed a written informed consent document, and the study protocol was approved by Peking University Health Science Center and University of Michigan’s institutional review board. All clinical investigation was conducted according to the principles expressed in the Declaration of Helsinki.

### Clinical and laboratory data

Baseline laboratory tests assessed complete blood count, liver function, renal function, lipids, fasting glucose, and hemoglobin A1c. Venous blood was collected after a 12-hour overnight fast on the second day of admission and analyzed using standard laboratory methods at Peking University Third Hospital. Venous samples for measurement of plasma Interleukin-6 (IL-6), monocyte chemoattractant protein (MCP1), tumor necrosis factor (TNF), soluble intercellular adhesion molecule 1 (sICAM1), soluble vascular adhesion molecule 1 (sVCAM1), and highly sensitive C-reactive peptide (Hs-CRP) were collected immediately after coronary angiography. All samples were drawn into EDTA vacutainer tubes and were centrifuged at 4°C for 15 minutes at 1000g within 30 minutes of collection. The samples were stored at -80°C until further analysis to avoid freeze-thaw cycles. Plasma levels of IL-6, MCP1, TNF, sICAM1, sVCAM1, and Hs-CRP were measured by enzyme-linked immunosorbent assay (ELISA) per the manufacturer’s instructions (Zhongshang Boao Inc., Beijing, China). MPO was quantified in plasma samples using a commercial ELISA kit (CardioMPO kit, Prognostix, Cleveland, OH). An investigator blinded to sample sources performed these assays.

### Quantification of highly sensitive and specific stable products of oxidation using LC/MS oxidized amino acids in HDL

All plasma work-ups and hydrolysis procedures were carried out at 4°C and HDL (d = 1.063–1.210g/ml) was prepared from plasma using sequential ultracentrifugation. HDL proteins were precipitated and delipidated, and oxidized amino acids were isolated by solid-phase extraction from an acid hydrolysate of HDL proteins. Oxidized amino acids were quantified using isotopically labeled internal standards, ^13^C_6_ tyrosine, ^13^C_6_ 3-chlorotyrosine, and ^13^C_6_ 3-nitrotyrosine via liquid chromatography-electrospray ionization tandem mass spectrometry (LC-ESI-MS/MS) with multiple reaction monitoring and positive ion acquisition mode. Labeled precursor amino acid, ^13^C_9_^15^N_1_tyrosine, was added to monitor potential internal artifact formation and was found to be negligible [16, 22].

### CEC assessment

J774 murine macrophages were labeled with 2μCi/mL ^3^H cholesterol (Perkin Elmer, Waltham, MA) for 24 hours in the presence of ACAT inhibitor (Sandoz 58–035) and equilibrated overnight with 0.3mM 8-(4-chlorophenylthio)-cyclic AMP to induce ABCA1 expression. ApoB-depleted serum was obtained using PEG precipitation. 2.8% v/v ApoB-depleted serum was used as the efflux acceptor for 4 hours. Efflux was quantified via liquid scintillation and expressed as a percentage of total cell ^3^H-cholesterol content. All assays were performed in duplicate. Plasma free media and pooled plasma were applied as controls [16].

### Liquid chromatography-electrospray ionization tandem mass spectrometry analysis to identify HDL proteome

50 μg HDL protein was precipitated with 10% trichloroacetic acid and solubilized with 6M urea and digested overnight at 37°C with trypsin (1:20, wt/wt, trypsin/HDL protein). The tryptic digests were acidified with trifluoroacetic acid, dried in a vacuum, re-suspended in 0.1% formic acid, and desalted with Waters Sep-Pack C18 filter cartridges before LC-ESI-MS/MS analysis.

Tryptic digests of HDL proteins were chromatographically separated with a nano-capillary reverse phase column (Acclaim PepMap C18, 2 microns, 15 cm, Thermo Scientific, San Jose, CA) using a 0.1% formic acid/acetonitrile gradient at 300 nL/min. The eluant was subjected to MS/MS in the Orbitrap Fusion tribrid mass spectrometer (Thermo Scientific, San Jose, CA). MS^1^ scans were acquired at 120,000 resolution. Data-dependent collision-induced dissociation MS/MS spectra were acquired with the top speed option (3 seconds) following each MS^1^ scan.

### Peptide and protein identification

Proteins were identified by searching the data against the *Homo Sapiens* database (UniProtKB) using Proteome Discoverer (v1.4, Thermo Scientific, San Jose, CA). Search parameters included MS^1^ mass tolerance of 10 ppm and fragment tolerance of 0.7 Da. Two missed cleavages were allowed; carbamidomethylation of cysteine was considered a fixed modification, and oxidation of methionine and phosphorylation of serine, threonine, and tyrosine were considered potential modifications. The Percolator algorithm was used to discriminate between correct and incorrect spectrum identification. The false discovery rate (FDR) was calculated at the peptide level and peptides with cut off <1% FDR. Only proteins with at least one unique peptide identified and presented in all samples were included for further analysis. For each protein identified by MS/MS, the peptide index was calculated using the formula: [(peptides in CAD sample/total peptides) x (% of CAD samples with ≥ 2 peptides)]- [(peptides in non-CAD /total peptides ≥2 peptides) x (% of non-CAD samples ≥ 2 peptides)]. Student’s t-test was used to compare the spectral counts of unique peptides in CAD and non-CAD groups [18]. A p-value of 0.05 was considered significant for all tests.

### Statistical analysis

All variables were represented as the mean ±standard deviation (SD) or percentage (%) as appropriate. Paired t-tests or ANOVA tests were used to compare variables between groups. Pearson correlation coefficients were calculated for the continuous variables. Skewed variables were log transformed (ln), and *p* <0.05 was considered significant. All analyses were conducted using SPSS, version 24 (IBM, USA).

## Results

### Baseline demographic and laboratory characteristics of Chinese non-CAD and CAD subjects by HDL status

The four groups of patients—non-CAD with low HDL, CAD with low HDL, non-CAD with high HDL, and CAD with high HDL—did not differ in mean age, gender, BMI, blood pressure, or smoking status (see Table 1). Additionally, hemoglobin A1C, renal function (as measured by serum creatinine), and blood count were unchanged among CAD and non-CAD subjects. Levels of LDL, apolipoprotein B (ApoB), Lipoprotein(a), and triglycerides were not significantly different between the four groups (see Table 2). The total cholesterol, HDL, and apolipoprotein A1 levels differed appropriately between the four groups per the study design. Levels of IL-6, MCP1, TNF, sICAM1, sVCAM1, and Hs-CRP were not different at this study’s baseline (see S1 Table).

**Table 1:** Demographic characteristics of the controls and coronary artery disease patients.

	Non CAD low HDL (n = 20)	CAD low HDL (n = 20)	Non CAD high HDL (n = 20)	CAD high HDL (n = 20)	P value
Age (years)	58.25±7.11	59.85±8.52	59.3±8.2	62.75±7.21	0.31
Male (%)	45%	60%	50%	65%	0.58
Waist Circumference (cm)	94.35±8.27	95.25±7.06*	87.25±8.67*	90.3±10.21	0.02
Body mass Index	**26.76±3.53**^**%**^	26.16±3.43*	23.02±2.56*^%^	24.55±3.52	0.002
Systolic Blood Pressure (mmHg)	123.25±10.17	125±11.92	124.05±11.91	127.25±13.42	0.74
Diastolic Blood Pressure (mmHg)	74.25±6.13	76.75±6.54	76.75±7.99	79.5±8.41	0.17
Current Smokers (%)	25%	25%	30%	55%	0.14
Remote Smokers (%)	35%	60%	40%	65%	0.16

CAD, coronary artery disease; Non CAD, healthy controls; HDL, high density lipoprotein.

Continuous and categorical variables are reported as mean ± standard deviation and percentages (%) respectively. Significant differences are represented as bold font (p<0.05).

*CAD low HDL vs. Non CAD high HDL; ^%^ Non CAD low HDL vs. Non CAD high HDL.

**Table 2:** Laboratory characteristics for the controls and coronary artery disease patients with low and high high density lipoprotein levels.

	Non CAD low HDL (n = 20)	CAD low HDL (n = 20)	Non CAD high HDL (n = 20)	CAD high HDL (n = 20)	p value
Hematocrit (Vol %)	40.37±3.6	41.23±3.45	42.25±3.93	39.31±10.48	0.48
White Blood Cells (10^9^/L)	6.09±1.61	6.29±1.22	5.8±1.7	6.53±1.44	0.47
Serum Creatinine (μmol)	74.85±13.89	76.1±16.39	80.7±11.45	80.25±18.92	0.54
Fasting Glucose (mmol)	4.96±0.55	5.02±0.77	5.1±0.65	5.13±0.84	0.87
Hemoglobin A1C (%)	5.62±0.41	5.69±0.44	5.5±0.33	5.67±0.41	0.37
Total Cholesterol (mmol)	4.53±0.89	**4.32±0.72**^**#**^	4.84±0.78	**5.18±1.06**^**#**^	0.02
LDL-C (mmol)	2.77±0.77	2.68±0.64	2.6±0.75	2.99±0.88	0.44
HDL-C (mmol)	**0.98±0.14**^**%@**^	0.90±0.15*^#^	1.58±0.25*^%^	**1.49±0.15**^**#@**^	0.00
Triglycerides (mmol)	1.72±0.72	1.78±0.99	1.31±0.98	1.53±0.89	0.35
Apolipoprotein B (mg/L)	887.55±236.71	886.6±182.17	765.9±233.79	942.35±271.61	0.12
Apolipoprotein A1 (mg/L)	**1320.6±169.53**^**%@**^	1176.7±201.28*^#^	1979.8±293.12*^%^	**1929.35±307.83**^**#@**^	0.00
*hs*-CRP (mg/L)	4.8±4.03	7.24±4.60	8.73±6.72	5.07±3.61	0.04

CAD, coronary artery disease; Non CAD, healthy controls; HDL, high density lipoprotein; LDL, low density Lipoprotein; hs-CRP, Highly sensitive- C-reactive Protein. Continuous variables are reported as mean ± standard deviation. Significant differences are represented as bold font (p<0.05) and differences between groups represented as % for Non CAD low HDL vs. Non CAD high HDL; @ for Non CAD low HDL vs. CAD high HDL; * for CAD low HDL vs. Non CAD high HDL; # for CAD low HDL vs. CAD high HDL

### CEC is decreased in Chinese CAD subjects

**Fig 1:**
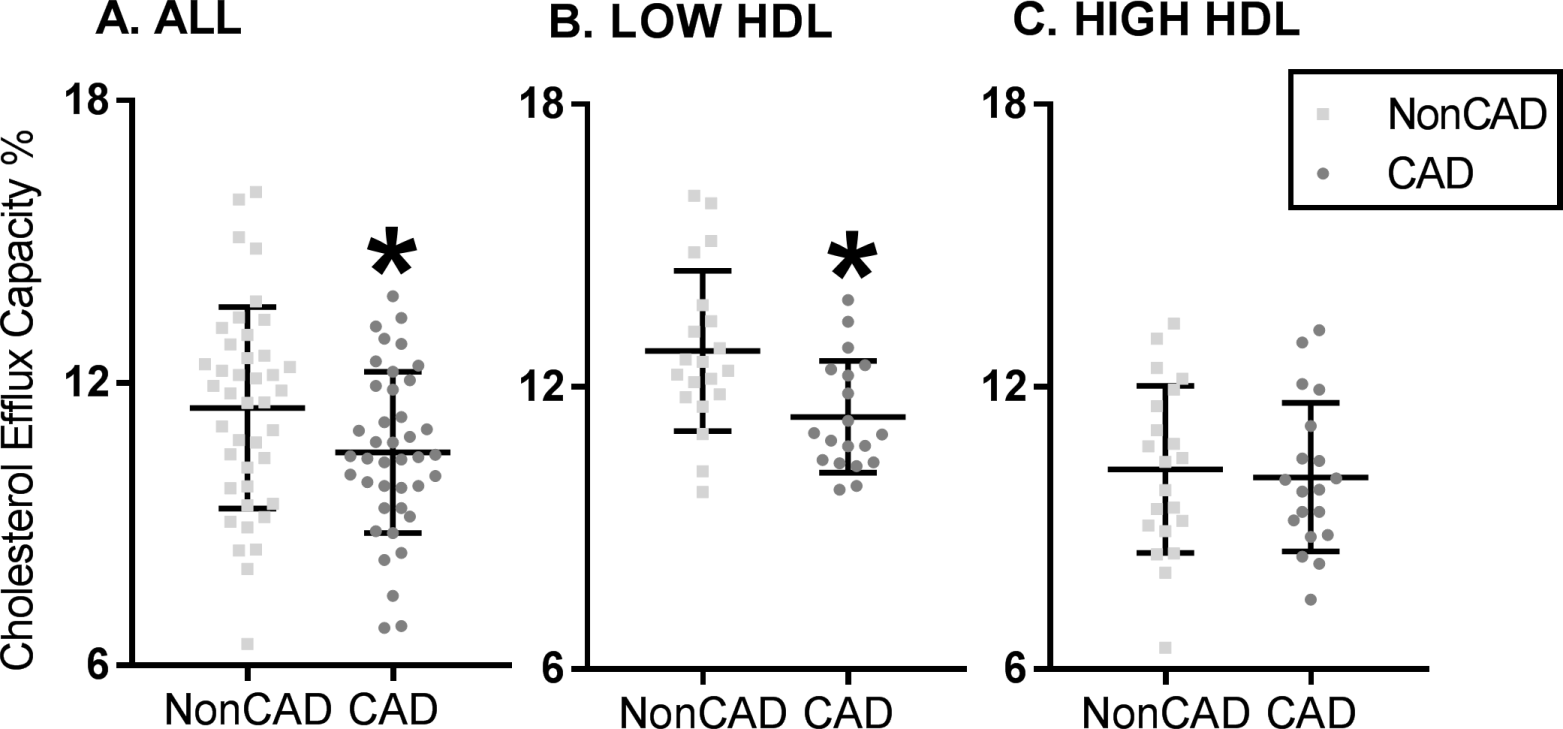
Cholesterol efflux capacity is decreased in coronary artery disease patients in China. (A) Cholesterol efflux capacity (CEC) in coronary artery disease (CAD) subjects compared to healthy controls (Non CAD) cohort in the entire cohort. (B) CEC shows differences in the CAD and non CAD cohort in the Low HDL group only and (C) shows differences in the high HDL cohort only. CEC is decreased in CAD group overall and specifically in the low HDL cohort, even as the high HDL cohort showed no differences between the CAD and non CAD group (*p value<0.05).

Recent studies suggest that HDL’s ability to accept cholesterol from J774 macrophages better identifies human CVD subjects compared to HDL level alone. Non-Apo B plasma CEC differed between the four groups (*p* <0.05) as shown in Fig 1. Specifically, there was a significant difference between the CAD (10.52±1.71) and non-CAD groups (11.42±2.14, *p* = 0.04; Fig 1A). CEC was significantly decreased in the CAD group (11.13±1.53 vs. 12.76±1.70; *p* = 0.003) in low-HDL subjects only (see Fig 1B). For high-HDL subjects, there was no difference between the CAD and non-CAD groups (see Fig 1C).

### HDL proteome is unaltered in Chinese CAD subjects

**Fig 2:**
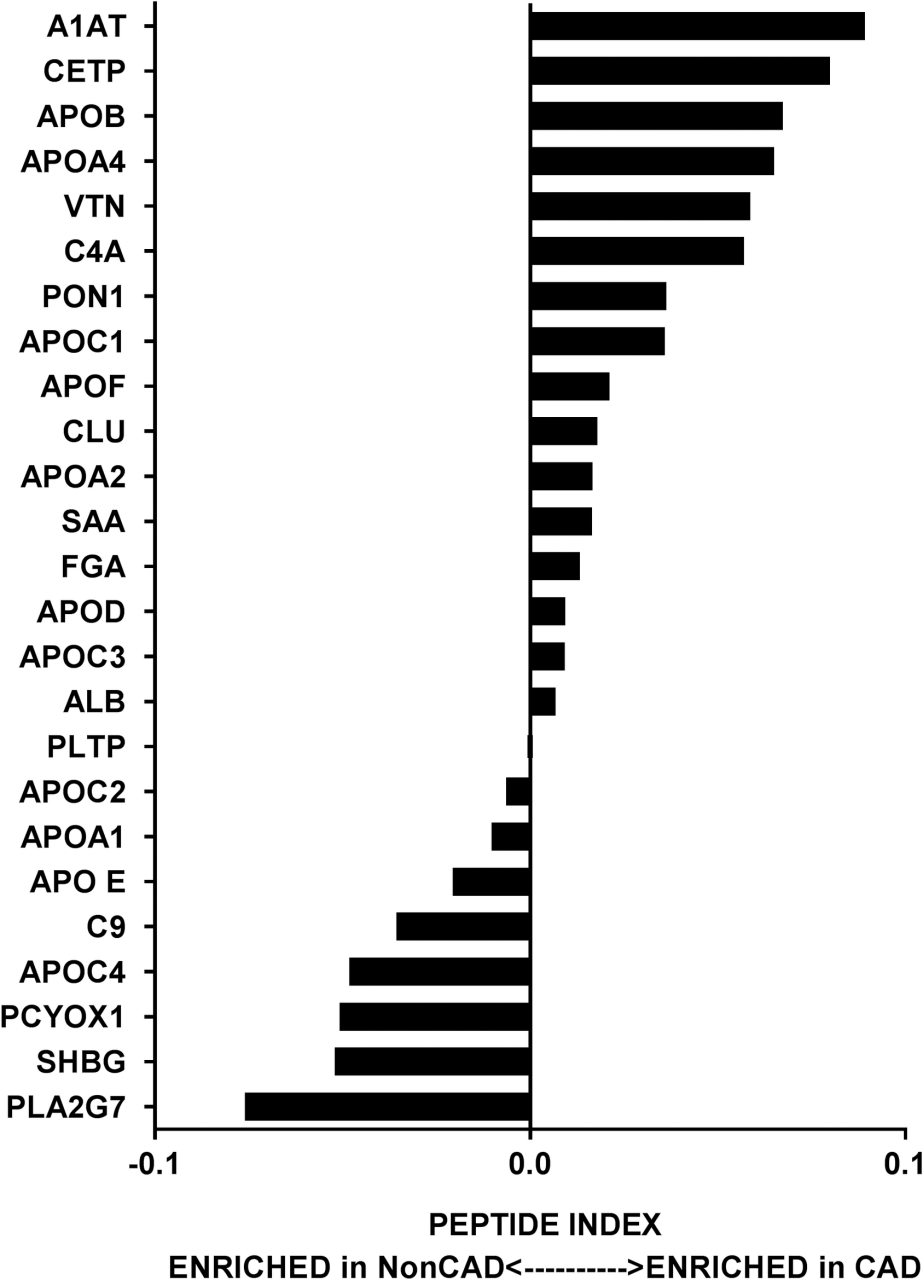
High-density lipoprotein protein composition is not altered in Chinese coronary artery disease subjects irrespective of high-density lipoprotein levels. The relative enrichment of 25 proteins in the high density lipoprotein (HDL) fraction of with and without coronary artery disease (CAD, Non CAD; n = 10 each) is represented by peptide index as described in the Methods section. No significant changes were observed between CAD and Non CAD subjects in both the cohorts. ALB-Albumin; A1AT-Alpha 1 antitrypsin; APO E-Apolipoprotein E; APOA1-Apolipoprotein A-I; APOA2-Apolipoprotein A-II; APOA4-Apolipoprotein A-IV; APOB-Apolipoprotein B; APOC1-Apolipoprotein C1; APOC2-Apolipoprotein C-II; APOC3-Apolipoprotein C3; APOC4- Apolipoprotein C-IV; APOD- Apolipoprotein D; APOF-Apolipoprotein F; C4A-Complement fragment 4A; C9-Complement 3; CETP-Cholesterol Ester transfer protein; CLU-Clusterin; FGA-Fibrinogen alpha; PCYOX1-Prenylcysteine oxidase 1; PLA2G7-Platelet-Activating Factor Acetylhydrolase; PLTP-Phospholipid transfer protein; PON1-Serum paraoxonase/arylesterase 1; SAA-Serum Amyloid A protein; SHBG- Sex hormone-binding globulin; VTN-Vitronectin.

We performed a targeted proteomic analysis of HDL to determine its protein cargo in all four groups. We used shotgun proteomics—direct analysis of a complex mixture of proteins—to study HDL [18]. After digesting the 50μg lipoprotein with trypsin, we analyzed the resultant peptide mixture with MS and matched the tandem mass spectra of the peptides with spectra in a protein database. This approach did not identify any enriched proteins (see Fig 2; S1 Fig). These data suggest that HDL’s protein composition is not a major driver of CAD in the Chinese population. Raw data is presented in S2 Table.

### MPO-specific chlorination is unchanged in Chinese CAD subjects

**Fig 3:**
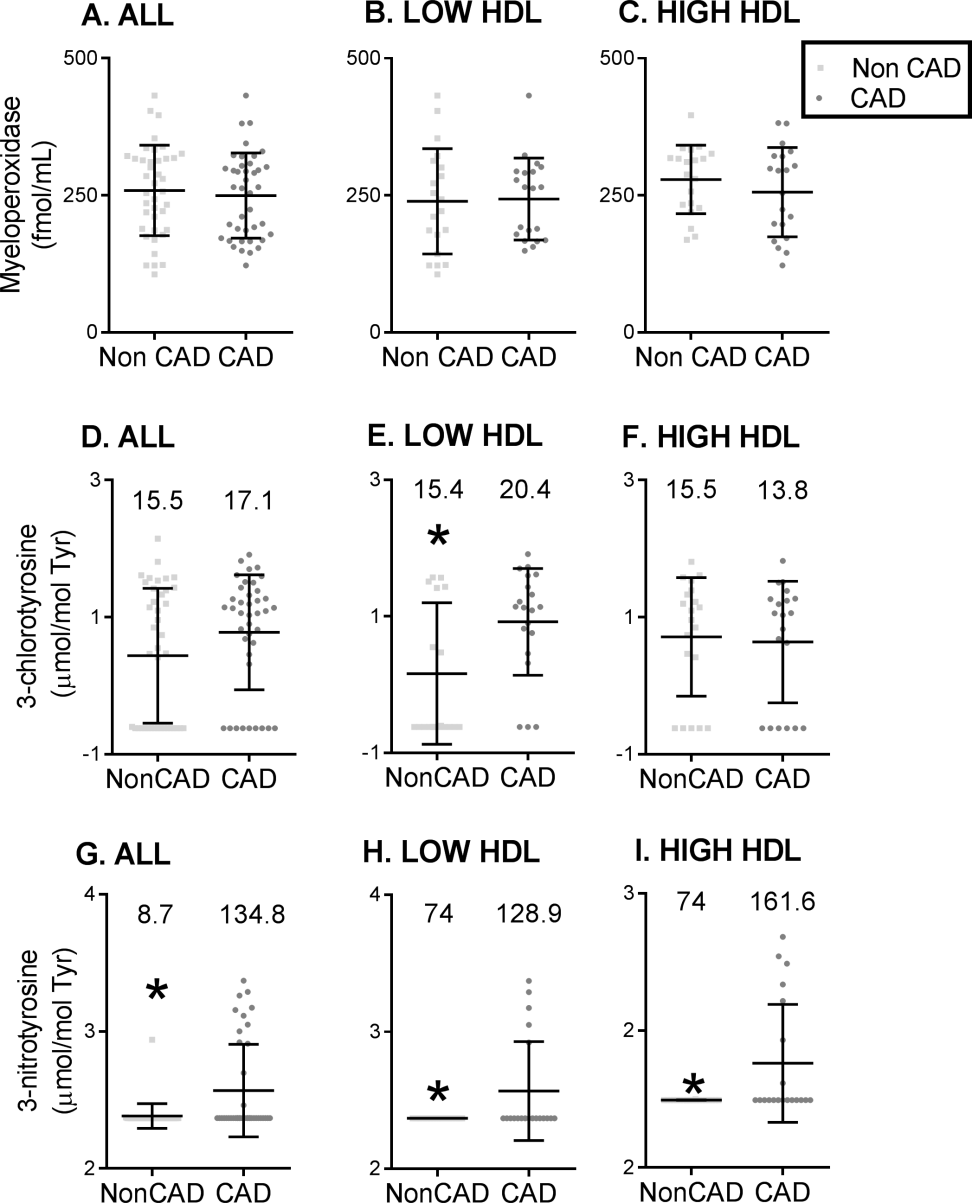
Plasma myeloperoxidase (MPO) levels and activity are unchanged in coronary artery disease patients in China. Plasma MPO levels in subjects with coronary artery disease (CAD) and without CAD (Non CAD) are represented by box plots of (A) All subjects (n = 40/group), (B) Low HDL subjects alone (n = 20/group) and (C) High HDL subjects alone (n = 20/group). MPO activity is represented by high density lipoprotein (HDL) 3-chlorotyrosine and 3-nitrotyrosine levels normalized to tyrosine levels determined by tandem mass spectrometry. Box plots display the distributions of HDL levels (log scale; log) of 3-chlorotyrosine in (D) All subjects (n = 40/group), (E) Low HDL subjects only (n = 20/group) and (F) High HDL subjects only (n = 20/group) between CAD and Non CAD groups. HDL 3-nitrotyrosine levels represented by box plot (log scale, log) of (G) All subjects (n = 40/group), (H) Low HDL subjects only (n = 20/group), and (I) High HDL subjects only (n = 20/group) between CAD and Non CAD groups. The plasma levels of MPO were not different between CAD and Non CAD subjects in the different cohorts. The HDL 3 chlorotyrosine levels were not different between groups except in the Low HDL subjects were the HDL 3-chlorotyrosine levels were elevated in the CAD subjects than in the non CAD subjects. The HDL 3-nitrotyrosine levels were elevated in the CAD subjects in the entire cohort and in both the low and high HDL subjects. The length of the box defines the interquartile range (IQR). Medians are reported for each group on the raw scale above the respective bar graphs. *denotes p<0.05.

There were no differences in plasma MPO levels among the four groups (see Fig 3A–3C). To quantify MPO activity, we measured specific MPO oxidation products in the HDL fraction: 3-chlorotyrosine and 3-nitrotyrosine. HDL from subjects with CAD (n = 40) exhibited significantly higher 3-nitrotyrosine content, but not 3-chlorotyrosine, compared to 40 non-CAD subjects (see Fig 3D and 3G). Because chlorination is the specific change attributed to MPO, whereas nitration can be derived from MPO or other RNS, these findings suggest that MPO may not cause HDL oxidation in CAD subjects; rather, RNS (not MPO) may be the potent oxidant in Chinese subjects.

To further evaluate the clinical spectrum of oxidized HDL, we compared the level of HDL oxidation in low- and high-HDL subjects with and without CAD. The HDL from all CAD subjects (irrespective of HDL levels) had significantly higher 3-nitrotyrosine content, implying that RNS-mediated oxidation is the primary driver of HDL oxidation in the PUHSC cohort. In contrast, 3-chlorotyrosine was increased in the low-HDL CAD group, suggesting that MPO oxidation plays a role in this subgroup only (see Fig 3E, 3F, 3H and 3I).

### HDL nitration does not correlate with chlorination

Plasma MPO levels did not correlate with levels of HDL 3-chlorotyrosine or 3-nitrotyrosine, suggesting they may not be derived from the vascular source and could be derived from an extravascular source (see S3 Table). To determine if chlorinated and nitrated species of HDL were correlated, we performed correlation of the entire cohort. As shown in S4 Table, chlorination did not correlate with nitration. Given that chlorination is a specific marker of MPO, this suggests that nitration occurs by an alternate mechanism independent of MPO. CEC did not correlate with 3-chlorotyrosine or 3-nitrotyrosine levels in the entire dataset (CAD, non-CAD, high-HDL, and low-HDL groups; see S5 Table). Therefore, the data demonstrate that MPO mediated-HDL oxidation is not responsible for HDL dysfunction in the Chinese cohort.

## Discussion

Our study highlights for the first time important ethnic differences between US and Chinese CAD cohorts. Like US CAD patients, Chinese patients demonstrated functional impairment of HDL resulting in diminished CEC in the CAD group. The data support the ‘dysfunctional HDL’ hypothesis but point to different mechanisms behind dysfunction in the Chinese cohort. HDL 3-nitrotyrosine was identified as the best predictive marker associated with CAD in the Chinese cohort irrespective of HDL levels, whereas HDL 3-chlorotyrosine appeared to be associated with CAD only in the low-HDL cohort. However, plasma MPO, HDL 3-nitrotyrosine, and 3-chlorotyrosine did not correlate with each other, suggesting that circulating MPO does not generate either of the two oxidative tyrosine modifications. The decreased CEC in the CAD group was unrelated to 3-nitrotyrosine levels, indicating that HDL dysfunction was related to neither MPO nor RNS. This result contrasts with the US CAD cohort, wherein subjects exhibited elevated MPO oxidation products associated with decreased CEC [22, 23]. Furthermore, unlike the US cohort [18], there were no differences in HDL proteome composition or circulating inflammatory markers or cytokines in the Chinese patients with CAD compared to their matched controls.

Since publication of the seminal paper on the Framingham cohort describing the inverse relationship between low HDL levels and cardiovascular risk [6], low HDL levels have become a secondary target of anti-lipidemic therapy after LDL reduction. Newer studies have questioned this association, as genetic variants that increase HDL levels do not confer cardiovascular protection [26]. Furthermore, recent therapeutic attempts to raise HDL levels have been disappointing and challenge the HDL hypothesis [27–29]. The predictive power of HDL levels in the Western and Chinese population is well known, but HDL levels have not been found to predict CAD severity in certain Chinese subjects [5, 9, 30]. Accumulating evidence points to HDL dysfunction rather than HDL mass as more predictive of HDL-mediated CVD risk. Our study was designed to explore the relationship between CAD risk and HDL dysfunction by recruiting well-matched CAD and non-CAD cohorts with HDL levels across the entire spectrum. The subjects were matched on age and gender as well as, most importantly, LDL levels. This makes the study design unique in its ability to examine the differences and mechanisms related to HDL function exclusively.

Decreased CEC has been shown to predict cardiovascular events in both US and Chinese populations. CEC improved CVD prediction in addition to the traditional cardiovascular risk factors in a large Chinese cohort [31]. Similarly, CEC in newly diagnosed CAD patients with a three year- follow-up confirmed the inverse relationship between CEC and cardiovascular mortality. Like our study, there was no association between CEC and HDL levels, and low CEC predicted endpoints of cardiovascular mortality independent of HDL levels [32]. The present study determined that efflux capacity was depressed in the CAD cohort when compared to the non-CAD cohort. In the low-HDL cohort, the CAD group had lower CEC compared to the non-CAD group; this finding was distinct from the high-HDL cohort, which showed no differences in efflux between the CAD and non-CAD group. While US CAD patients with very high HDL (mean 86mg/dL) had decreased efflux compared to HDL-matched controls, our cohort did not demonstrate the same effect [33]. This evidence confirms that low HDL mass in CAD patients downregulated CEC which increases CVD risk.

Cytokines and inflammatory markers have been linked to acute CAD in Western cohorts [34, 35]. A similar exploration of a Chinese CAD cohort revealed a correlation of inflammatory markers associated with CAD severity [36]. We did not find any significant changes in the inflammatory markers and cytokines in our Chinese cohort to explain the differences in HDL mass or CAD risk. The HDL proteome is dynamic and responsive to inflammation and oxidative stress. Chinese CAD patients demonstrated decreased efflux and altered HDL protein composition when compared to controls despite similar HDL levels and different LDL levels [37]. Similarly, Yan et al. used isobaric tag for relative and absolute quantitation labeling to identify 12 proteins differentially regulated in Chinese CAD patients compared to controls. The upregulated proteins were involved in acute phase change, inflammatory response, and platelet activation whereas the downregulated proteins were involved in lipid metabolism [38]. HDL proteome in US CAD patients has been shown to be altered and enriched with inflammatory proteins and lacking in anti-inflammatory and anti-oxidative proteins [39–41]. In our study, HDL proteomic assessment did not yield any differentially regulated proteins, indicating that proteome changes are not the mechanism behind the HDL dysfunction which accompanies CAD.

In US cohorts, MPO levels predict cardiovascular risk, events, and burden and alter the key HDL protein, apolipoprotein A1. MPO levels are associated with CAD risk in Chinese type 2 diabetics [42]; however, MPO levels measured in our cohort did not demonstrate any changes between the four groups. MPO activity products, namely 3-chlorotyrosine and 3-nitrotyrosine were elevated and decreased efflux in Chinese type 2 diabetic patients [43]. In our cohort, 3-chlorotyrosine did not associate with CAD, indicating no relationship between MPO action and CAD in the Chinese patients except in the CAD low-HDL subgroup. 3-nitrotyrosine was elevated in CAD patients in the high- and low-HDL groups, although 3-nitrotyrosine and MPO-specific 3-chlorotyrosine were not correlated, indicating that 3-nitrotyrosine is derived from other RNS and not from MPO action. CEC function, which was depressed in the CAD group, was not associated with 3-chlorotyrosine or 3-nitrotyrosine levels in the entire dataset regardless of HDL and CAD status. These results indicate that mechanisms of CAD may be distinct in diabetic subjects as opposed non-diabetics which was the focus of our investigation. HDL dysfunction in the non-diabetic Chinese cohort was not MPO-mediated; thus, the mechanism behind this dysfunction warrants further exploration.

While there were no overall differences in MPO oxidation of HDL in the Chinese CAD and non-CAD subjects, in subgroup analysis, MPO oxidation (3-chlorotyrosine) was increased in the CAD group of low-HDL subjects. Hence, MPO may play a role in this subgroup, but there was no correlation between 3-chlorotyrosine and CEC. Further research is necessary to understand why low-HDL CAD subjects appear vulnerable to MPO-mediated oxidative damage and to uncover alternate mechanisms by which, despite unchanged MPO levels, MPO oxidizes HDL in the low-HDL CAD cohort. The subtle decrease in CEC and increase in chlorotyrosine in the CAD low-HDL subgroup indicates HDL dysfunction, though the relationship between chlorotyrosine and CEC is not apparent in the correlation analysis. Chinese subjects with CAD exhibited decreased efflux but increased MPO oxidation in the CAD low-HDL group, possibly from extravascular sources.

Our study had many limitations, including a small sample size, and because of the definite HDL selection criterion, the results can only be extrapolated to the extremes of HDL mass. Extensive matching of different factors like age, gender, HDL, and LDL status in these cohorts might have introduced some unknown selection bias. Preliminary studies have suggested that Chinese CAD subjects’ HDL is dysfunctional in several ways [44]. HDL can also exhibit altered lipid composition [45] that causes decreased efflux in US CAD patients [33]. Altered lipid composition may mediate dysfunctional HDL in Chinese subjects as well, while protein changes and MPO may not play important roles. This notion requires further exploration via mechanistic studies. While speculative, several potential mechanisms that could explain the observed differences between Chinese and US cohorts. First, environmental factors like higher smoking rates [46, 47] and poor air quality [48], could be contributing to the to the unique cardiovascular risk in Chinese population [48]. For example, smoking and air pollution can affect atherogenesis independent of lipoprotein oxidation and dysfunction such as altered vascular reactivity and immunomodulation amongst others [49]. Second, our study focused on myeloperoxidase mediated HDL oxidation, as this mechanism has been extensively studied in multiple US cohorts. There might be alternate pathways of oxidation which were not explored in this study such as nonenzymatic oxidation with free metal ions (hydroxyl radical) and glycation. Enzymatic processes such as NADPH oxidase, endothelial nitric oxide synthase (eNOS) uncoupling, and matrix metalloproteases could be the other sources of oxidation in the Chinese cohorts (reviewed in [50–52]). Similarly, our work focuses on oxidation of tyrosine residues as this modification has been the most reported in the literature. Oxidation of other residues such as tryptophan [53, 54] and methionine [55] in apolipoprotein A1 could also be involved but was not measured. Third, the HDL fraction in human plasma is heterogeneous, consisting of several subpopulations of particles of varying sizes. HDL particle size could also alter HDL function but were not measured in this study [56]. Fourth, our work used CEC as a metric of HDL function, as this is the most extensively studied and shown in multiple large clinical trials in the US cohorts to be predictive of cardiovascular events [12, 13]. However, there might be other aspects of HDL function that might have correlated better with oxidative stress which were not explored in this work. These include inhibition of vascular inflammation and thrombosis, enhancement of endothelial repair, angiogenesis and endothelial function [57]. Finally, unique genetic variations in lipoprotein biology [58–60] could contribute differentially to cardiovascular risk in Chinese subjects compared with US cohorts.

In conclusion, our study highlighted unique differences in the pathophysiology of Chinese patients with CAD. These subjects exhibited diminished CEC, specifically in the low-HDL CAD group. We found no changes to HDL proteome and MPO levels, but there was evidence of elevated 3-chlorotyrosine levels in the CAD low-HDL cohort. Nitrotyrosine was elevated in CAD patients irrespective of HDL status, but it did not correlate with chlorotyrosine, suggesting non-MPO-mediated nitration of HDL. Taken together, these changes point to unique mechanisms in the Chinese cohort promoting HDL dysfunction in CAD; hence, further exploration of the mechanisms behind HDL dysfunction in the Chinese population is warranted.

## Supporting information

S1 Fig High density lipoprotein protein composition is not altered in Chinese CAD subjects irrespective of high density lipoprotein levels. The relative enrichment of 25 proteins in the high density lipoprotein (HDL) fraction of with and without coronary artery disease (CAD, Non CAD; n = 10 each) in both (A) Low HDL cohort and (B) High HDL cohort is represented by peptide index as described in the Methods section. No significant changes were observed between CAD and Non CAD subjects in both the HDL cohorts. ALB-Albumin; A1AT-Alpha 1 antitrypsin; APO E-Apolipoprotein E; APOA1-Apolipoprotein A-I; APOA2-Apolipoprotein A-II; APOA4-Apolipoprotein A-IV; APOB-Apolipoprotein B; APOC1-Apolipoprotein C1; APOC2-Apolipoprotein C-II; APOC3-Apolipoprotein C3; APOC4- Apolipoprotein C-IV; APOD- Apolipoprotein D; APOF-Apolipoprotein F; C4A-Complement fragment 4A; C9-Complement 3; CETP-Cholesterol Ester transfer protein; CLU-Clusterin; FGA-Fibrinogen alpha; PCYOX1-Prenylcysteine oxidase 1; PLA2G7-Platelet-Activating Factor Acetylhydrolase; PLTP-Phospholipid transfer protein; PON1-Serum paraoxonase/arylesterase 1; SAA-Serum Amyloid A protein; SHBG- Sex hormone-binding globulin; VTN-Vitronectin.
(DOCX)

S1 Table Cytokine levels in the four groups. (DOCX)

S2 Table Protein spectral counts of the high-density lipoprotein proteome reveals no changes in coronary artery disease subjects compared with non-coronary artery disease subjects. (XLSX)

S3 Table Correlations of myeloperoxidase oxidation products with myeloperoxidase levels. (DOCX)

S4 Table Correlation of high-density lipoprotein 3-chlorotyrosine to 3-nitrotyrosine levels. (DOCX)

S5 Table Correlation analysis of cholesterol efflux capacity with oxidation markers. (DOCX)
