# Supplementary material for: Myeloperoxidase mediated HDL oxidation and HDL proteome changes do not contribute to dysfunctional HDL in Chinese subjects with coronary artery disease
Source: PLoS One. 2018 Mar 5;13(3):e0193782. doi: 10.1371/journal.pone.0193782 (PMC5837105; doi:10.1371/journal.pone.0193782)
Supplement: S1 Fig — The relative enrichment of 25 proteins in the high density lipoprotein (HDL) fraction of with and without coronary artery disease (CAD, Non CAD; n = 10 each) in both (A) Low HDL cohort and (B) High HDL cohort is represented by peptide index as described in the Methods section. No significant changes were observed between CAD and Non CAD subjects in both the HDL cohorts. ALB-Albumin; A1AT-Alpha 1 antitrypsin; APO E-Apolipoprotein E; APOA1-Apolipoprotein A-I; APOA2-Apolipoprotein A-II; APOA4-Apolipoprotein A-IV; APOB-Apolipoprotein B; APOC1-Apolipoprotein C1; APOC2-Apolipoprotein C-II; APOC3-Apolipoprotein C3; APOC4- Apolipoprotein C-IV; APOD- Apolipoprotein D; APOF-Apolipoprotein F; C4A-Complement fragment 4A; C9-Complement 3; CETP-Cholesterol Ester transfer protein; CLU-Clusterin; FGA-Fibrinogen alpha; PCYOX1-Prenylcysteine oxidase 1; PLA2G7-Platelet-Activating Factor Acetylhydrolase; PLTP-Phospholipid transfer protein; PON1-Serum paraoxonase/arylesterase 1; SAA-Serum Amyloid A protein; SHBG- Sex hormone-binding globulin; VTN-Vitronectin. (DOCX) [file pone.0193782.s001.docx]

| **Fig S1. High-density lipoprotein protein composition is not altered in Chinese coronary artery**  **disease subjects irrespective of high-density lipoprotein levels.**   |  |  |  |
| --- | --- | --- | --- |

The relative enrichment of 25 proteins in the high density lipoprotein (HDL) fraction of with and without coronary artery disease (CAD, Non CAD; n=10 each) in both (A) Low HDL cohort and (B) High HDL cohort is represented by peptide index as described in the Methods section. No significant changes were observed between CAD and Non CAD subjects in both the HDL cohorts. ALB-Albumin; A1AT-Alpha 1 antitrypsin; APO E-Apolipoprotein E; APOA1-Apolipoprotein A-I ; APOA2-Apolipoprotein A-II; APOA4- Apolipoprotein A-IV; APOB-Apolipoprotein B; APOC1-Apolipoprotein C1; APOC2-Apolipoprotein C-II; APOC3-Apolipoprotein C3; APOC4- Apolipoprotein C-IV; APOD- Apolipoprotein D; APOF-Apolipoprotein F; C4A-Complement fragment 4A; C9-Complement 3; CETP-Cholesterol Ester transfer protein; CLU-Clusterin; FGA-Fibrinogen alpha; PCYOX1-Prenylcysteine oxidase 1; PLA2G7-Platelet-Activating Factor Acetylhydrolase; PLTP-Phospholipid transfer protein; PON1-Serum paraoxonase/arylesterase 1; SAA-Serum Amyloid A protein; SHBG- Sex hormone-binding globulin; VTN-Vitronectin.
