# Supplementary material for: Myeloperoxidase mediated HDL oxidation and HDL proteome changes do not contribute to dysfunctional HDL in Chinese subjects with coronary artery disease
Source: PLoS One. 2018 Mar 5;13(3):e0193782. doi: 10.1371/journal.pone.0193782 (PMC5837105; doi:10.1371/journal.pone.0193782)
Supplement: S1 Table — (DOCX) [file pone.0193782.s002.docx]

| **S1 Table. Cytokine levels in the four groups.** | | | |  |  |
| --- | --- | --- | --- | --- | --- |
|  |  |  |  |  |  |
| **Variables (units)** | **Non CAD low HDL**  **(n=20)** | **CAD low HDL (n=20)** | **Non CAD high HDL (n=20)** | **CAD high HDL**  **(n=20)** | **p value** |
| Interleukin-6 (pg/mL) | 2.46±2.51 | 2.89±1.62 | 4.81±4.32 | 2.79±2.40 | 0.05 |
| Monocyte Chemoattractant Protein 1 (pg/mL) | 123.94±64.33 | 112.58±39.12 | 149.42±72.25 | 126.82±41.28 | 0.22 |
| Tumor Necrosis Factor α (pg/mL) | 11.33±26.1 | 5.46±2.86 | 31.1±92.88 | 6.14±3.03 | 0.3 |
| Soluble Intercellular Adhesion Molecule 1 (ng/mL) | 407.69±235.07 | 395.71±160.05 | 488.25±242.61 | 429.54±170.58 | 0.5 |
| Soluble Vascular Adhesion Molecule 1 (ng/mL) | 1180.47±667.3 | 1509.42±758.0 | 1720.75±963.3 | 1305.09±752.9 | 0.16 |
