# Supplementary material for: Myeloperoxidase mediated HDL oxidation and HDL proteome changes do not contribute to dysfunctional HDL in Chinese subjects with coronary artery disease
Source: PLoS One. 2018 Mar 5;13(3):e0193782. doi: 10.1371/journal.pone.0193782 (PMC5837105; doi:10.1371/journal.pone.0193782)
Supplement: S3 Table — (DOCX) [file pone.0193782.s004.docx]

| \| **S3 Table. Correlations of myeloperoxidase oxidation products with myeloperoxidase levels.** \| \| \| \| \| --- \| --- \| --- \| --- \| \|  \| **Pearson Correlation** \| **Sig. (2-tailed)** \| **N** \| \| **All** \|  \|  \|  \| \| 3-Chlorotyrosine \| -0.082 \| 0.469 \| 80 \| \| 3-Nitrotyrosine \| -0.015 \| 0.895 \| 80 \| \| **Non-CAD** \|  \|  \|  \| \| 3-Chlorotyrosine \| -0.137 \| 0.399 \| 40 \| \| 3-Nitrotyrosine \| -0.227 \| 0.159 \| 40 \| \| **CAD** \|  \|  \|  \| \| 3-Chlorotyrosine \| -0.089 \| 0.585 \| 40 \| \| 3-Nitrotyrosine \| -0.057 \| 0.728 \| 40 \| \| **Low HDL** \|  \|  \|  \| \| 3-Chlorotyrosine \| -0.089 \| 0.586 \| 40 \| \| 3-Nitrotyrosine \| -0.025 \| 0.881 \| 40 \| \| **High HDL** \|  \|  \|  \| \| 3-Chlorotyrosine \| -0.066 \| 0.687 \| 40 \| \| 3-Nitrotyrosine \| -0.007 \| 0.968 \| 40 \| \| **Non-CAD Low HDL** \|  \|  \|  \| \| 3-Chlorotyrosine \| -0.341 \| 0.141 \| 20 \| \| 3-Nitrotyrosine \| -0.286 \| 0.221 \| 20 \| \| **CAD Low HDL** \|  \|  \|  \| \| 3-Chlorotyrosine \| 0.066 \| 0.783 \| 20 \| \| 3-Nitrotyrosine \| -0.054 \| 0.821 \| 20 \| \| **Non-CAD High HDL** \|  \|  \|  \| \| 3-Chlorotyrosine \| 0.143 \| 0.549 \| 20 \| \| 3-Nitrotyrosine \| 0.068 \| 0.775 \| 20 \| \| **CAD High HDL** \|  \|  \|  \| \| 3-Chlorotyrosine \| -0.245 \| 0.297 \| 20 \| \| 3-Nitrotyrosine \| -0.061 \| 0.799 \| 20 \| \| 3-Chlorotyrosine and 3-Nitrotyrosine per mmol of tyrosine  r per Pearson’s correlation \| \| \|  \| |  |  |  |
| --- | --- | --- | --- | --- | --- | --- | --- | --- | --- | --- | --- | --- | --- | --- | --- | --- | --- | --- | --- | --- | --- | --- | --- | --- | --- | --- | --- | --- | --- | --- | --- | --- | --- | --- | --- | --- | --- | --- | --- | --- | --- | --- | --- | --- | --- | --- | --- | --- | --- | --- | --- | --- | --- | --- | --- | --- | --- | --- | --- | --- | --- | --- | --- | --- | --- | --- | --- | --- | --- | --- | --- | --- | --- | --- | --- | --- | --- | --- | --- | --- | --- | --- | --- | --- | --- | --- | --- | --- | --- | --- | --- | --- | --- | --- | --- | --- | --- | --- | --- | --- | --- | --- | --- | --- | --- | --- | --- | --- | --- | --- | --- | --- | --- | --- | --- | --- | --- | --- | --- | --- | --- | --- | --- |
