# Supplementary material for: Myeloperoxidase mediated HDL oxidation and HDL proteome changes do not contribute to dysfunctional HDL in Chinese subjects with coronary artery disease
Source: PLoS One. 2018 Mar 5;13(3):e0193782. doi: 10.1371/journal.pone.0193782 (PMC5837105; doi:10.1371/journal.pone.0193782)
Supplement: S4 Table — (DOCX) [file pone.0193782.s005.docx]

| **S4 Table. Correlation of high-density lipoprotein 3-Chlorotyrosine to 3-Nitrotyrosine Levels.** | | | |
| --- | --- | --- | --- |
|  | **r** | **p value** | **N** |
| All subjects | 0.107 | 0.34 | 80 |
| CAD group | 0.017 | 0.91 | 40 |
| Non CAD group | 0.177 | 0.27 | 40 |
| Low HDL | 0.268 | 0.09 | 40 |
| High HDL | 0.096 | 0.55 | 40 |
| 3-Chlorotyrosine and 3-Nitrotyrosine per mmol tyrosine | | | |
| r per Pearson's correlation |  |  |  |

|  |
| --- |
