# Supplementary material for: Myeloperoxidase mediated HDL oxidation and HDL proteome changes do not contribute to dysfunctional HDL in Chinese subjects with coronary artery disease
Source: PLoS One. 2018 Mar 5;13(3):e0193782. doi: 10.1371/journal.pone.0193782 (PMC5837105; doi:10.1371/journal.pone.0193782)
Supplement: S5 Table — (DOCX) [file pone.0193782.s006.docx]

|  | | | | |  |  |  |
| --- | --- | --- | --- | --- | --- | --- | --- |
| **S5 Table. Correlation analysis of cholesterol efflux capacity with oxidation Markers.** | | | |  |  |  |  |
|  | **r** | **p value** | **N** |  |  |  |  |
| **3-Chlorotyrosine** |  |  |  |  |  |  |  |
| All comers | -0.074 | 0.513 | 80 |  |  |  |  |
| CAD group | -0.264 | 0.1 | 40 |  |  |  |  |
| Non CAD group | 0.124 | 0.447 | 40 |  |  |  |  |
| Low HDL | 0.112 | 0.493 | 40 |  |  |  |  |
| High HDL | -0.235 | 0.144 | 40 |  |  |  |  |
| **3-Nitrotyrosine** |  |  |  |  |  |  |  |
| All comers | -0.13 | 0.252 | 80 |  |  |  |  |
| CAD group | -0.138 | 0.396 | 40 |  |  |  |  |
| Non CAD group | 0.147 | 0.366 | 40 |  |  |  |  |
| Low HDL group | -0.236 | 0.143 | 40 |  |  |  |  |
| High HDL group | -0.074 | 0.652 | 40 |  |  |  |  |
| 3-Chlorotyrosine and 3-Nitrotyrosine per mmol of tyrosine | | | |  |  |  |  |
| r per Pearson's correlation | | | |  |  |  |  |
